# The effects of remote ischaemic preconditioning on coronary artery function in patients with stable coronary artery disease

**DOI:** 10.1016/j.ijcard.2017.10.082

**Published:** 2018-02-01

**Authors:** D. Corcoran, R. Young, P. Cialdella, P. McCartney, A. Bajrangee, B. Hennigan, D. Collison, D. Carrick, A. Shaukat, R. Good, S. Watkins, M. McEntegart, J. Watt, P. Welsh, N. Sattar, A. McConnachie, K.G. Oldroyd, C. Berry

**Affiliations:** a British Heart Foundation Glasgow Cardiovascular Research Centre, University of Glasgow, Scotland, UK; b West of Scotland Heart and Lung Centre, Golden Jubilee National Hospital, Glasgow, Scotland, UK; c Robertson Centre for Biostatistics, University of Glasgow, Scotland, UK

**Keywords:** ACh, acetylcholine, ANCOVA, analysis of covariance, ANOVA, analysis of variance, ADMA, asymmetric dimethylarginine, CONSORT, Consolidated Standards of Reporting Trials, CAESAR, Consortium for preclinicAl assESsment of cARdioprotective therapies, CAD, coronary artery disease, ECG, electrocardiogram, IMR, index of microcirculatory resistance, IL-6, interleukin-6, MPO, myeloperoxidase, MI, myocardial infarction, NO, nitric oxide, PCI, percutaneous coronary intervention, QCA, quantitative coronary angiography, RIPC, remote ischaemic preconditioning, t-PA, tissue plasminogen activator, vWF, Von Willebrand factor, Remote ischaemic preconditioning, Coronary artery disease, Endothelial function, Cardioprotection, Myocardial infarction

## Abstract

**Background:**

Remote ischaemic preconditioning (RIPC) is a cardioprotective intervention invoking intermittent periods of ischaemia in a tissue or organ remote from the heart. The mechanisms of this effect are incompletely understood. We hypothesised that RIPC might enhance coronary vasodilatation by an endothelium-dependent mechanism.

**Methods:**

We performed a prospective, randomised, sham-controlled, blinded clinical trial. Patients with stable coronary artery disease (CAD) undergoing elective invasive management were prospectively enrolled, and randomised to RIPC or sham (1:1) prior to angiography. Endothelial-dependent vasodilator function was assessed in a non-target coronary artery with intracoronary infusion of incremental acetylcholine doses (10^− 6^, 10^− 5^, 10^− 4^ mol/l). Venous blood was sampled pre- and post-RIPC or sham, and analysed for circulating markers of endothelial function. Coronary luminal diameter was assessed by quantitative coronary angiography. The primary outcome was the between-group difference in the mean percentage change in coronary luminal diameter following the maximal acetylcholine dose (Clinicaltrials.gov identifier: NCT02666235).

**Results:**

75 patients were enrolled. Following angiography, 60 patients (mean ± SD age 57.5 ± 8.5 years; 80% male) were eligible and completed the protocol (n = 30 RIPC, n = 30 sham). The mean percentage change in coronary luminal diameter was − 13.3 ± 22.3% and − 2.0 ± 17.2% in the sham and RIPC groups respectively (difference 11.32%, 95%CI: 1.2– 21.4, p = 0.032). This remained significant when age and sex were included as covariates (difference 11.01%, 95%CI: 1.01– 21.0, p = 0.035). There were no between-group differences in endothelial-independent vasodilation, ECG parameters or circulating markers of endothelial function.

**Conclusions:**

RIPC attenuates the extent of vasoconstriction induced by intracoronary acetylcholine infusion. This endothelium-dependent mechanism may contribute to the cardioprotective effects of RIPC.

## 1 Introduction

Ischaemic conditioning describes the beneficial effects of repeated cycles of ischaemia and reperfusion resulting in cardioprotection against ischaemia-reperfusion injury [1], [2], [3], [4], [5], [6]. This cardioprotective strategy may be applied either directly to the heart or remotely, and before or during ischaemia-reperfusion injury [7]. Remote ischaemic preconditioning (RIPC) has been investigated in several ischaemia-reperfusion injury clinical settings, including patients with stable coronary artery disease (CAD) undergoing elective percutaneous coronary intervention (PCI) and surgical revascularisation [8], [9], [10], [11], [12], [13], [14], [15]. RIPC is associated with a reduction in infarct size in patients following an acute myocardial infarction (MI) and after elective PCI in patients with stable CAD [8], [16], [17]. The mechanisms by which the cardioprotective effects of RIPC act are incompletely understood [18]. The effects of RIPC on health outcomes in patients with acute ST-segment elevation myocardial infarction (STEMI) are currently being assessed in two large phase 3 clinical trials in Europe (CONDI-2 NCT01857414 and ERIC NCT02342522).

Three components of the RIPC stimulus may be defined: signal generation from the tissue or organ remote from the heart, transmission of the cardioprotective signal to the heart, and the mechanism of the cardiac response [19]. The molecular mechanisms and signal transduction of the conditioning phenomena in the heart have been described as involving extracellular trigger molecules, intracellular protein kinase activation and the mitochondria as the end-effector [20]. The signal transduction from the distant organ/tissue to the heart is incompletely understood, but likely involves both neural and hormonal pathways [21], [22], [23], [24].

Since coronary artery function has pivotal importance for regulating myocardial perfusion, we hypothesised that RIPC would have favourable effects on coronary artery endothelial dysfunction. Endothelial dysfunction is prevalent in patients with atherosclerotic CAD and associated with adverse health outcomes [25], [26], [27]. This mechanism could potentially contribute to the cardioprotective effects of RIPC through enhanced coronary artery blood flow leading to improved myocardial perfusion following ischaemia-reperfusion injury. Studies of RIPC have identified endothelial-dependent responses that potentially implicate endothelial cell activation in mediating enhanced coronary blood flow and cardioprotective effects [28], [29], [30], [31]. Conversely, we are not aware of experimental evidence for an effect of RIPC on endothelial-independent vascular function.

Our first aim was to determine whether RIPC might affect coronary artery function in vivo in patients with stable CAD. Should this be the case, we next aimed to determine the contribution of endothelium-dependent and -independent function on the observed responses. Our second aim was to assess whether circulating molecules reflecting endothelial cell vasodilatory and fibrinolytic function might be associated with a RIPC-mediated effect on coronary artery function. Thirdly, since a neural hypothesis implicates activation of the autonomic nervous system via one or more pathways [32], [33], [34], we aimed to assess cardiac conduction using the surface electrocardiogram (ECG) [35].

## 2 Methods

### 2.1 Trial design

We performed a prospective, randomised, sham-controlled, blinded (physician, researcher) clinical trial.

### 2.2 Study population

Patients undergoing elective invasive coronary angiography for investigation of stable CAD were enrolled and provided written informed consent. The study was approved by the National Research Ethics Service (reference 10/S0704/52). The ClinicalTrials.gov identifier is NCT02666235. Patients were eligible if, following initial coronary angiography, there was a main epicardial coronary artery suitable for coronary reactivity testing (either an angiographically normal coronary artery, or an artery with minimal plaque burden and without an epicardial diameter stenosis ≥ 40%). Exclusion criteria were MI < 2 weeks, previous coronary artery bypass grafting, second or third degree atrioventricular block, and inability to provide informed consent.

### 2.3 Setting

The study took place between July 2011 and March 2016 in a regional cardiac centre. Potentially eligible participants were identified by screening clinically-indicated referrals for invasive coronary angiography.

### 2.4 Informed consent

Eligible patients were sent a Patient Information Sheet (PIS) before attending hospital for the clinically-indicated coronary angiogram. The PIS had been approved by the local ethics committee and written informed consent was obtained on the ward before the procedure.

### 2.5 Randomisation, implementation and blinding

Randomisation took place immediately after obtaining verbal consent using a web-based computer tool with a concealed random allocation sequence provided by the independent clinical trials unit and implemented by the researcher (D.C.). Randomisation was on a 1:1 basis between RIPC or sham immediately prior to the invasive procedure. The study was conducted according to CONSORT guidelines for clinical trials.

### 2.6 Intervention

#### 2.6.1 Remote ischaemic preconditioning

RIPC was performed according to a standard protocol involving intermittent inflation of an arm sphygmomanometer cuff for 5 min periods at 200 mm Hg, separated by a 5-minute rest interval, and repeated successively on 4 occasions [8].

#### 2.6.2 Sham procedure

The sham procedure involved cuff placement alone without inflation.

Following the RIPC or sham procedure, patients underwent angiography and coronary reactivity testing within 1 h.

### 2.7 Primary and secondary endpoints

**Fig. 1:**
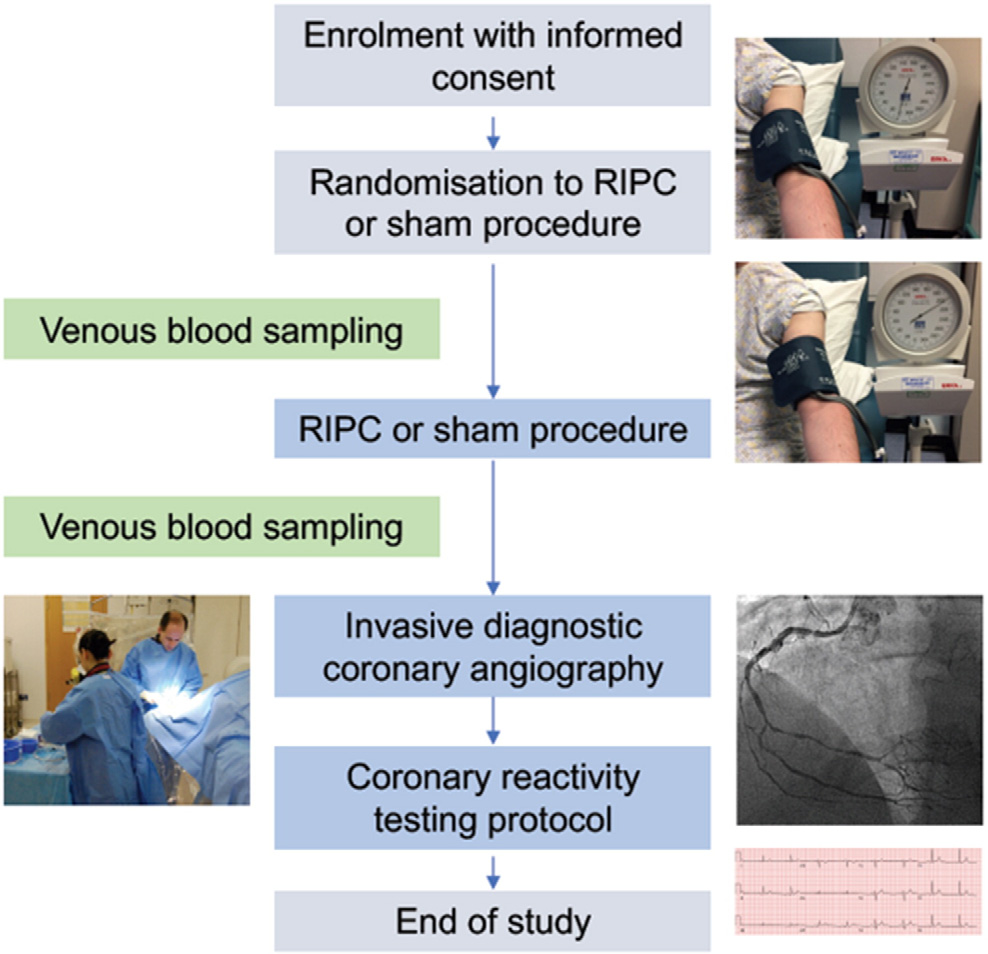
Illustration of the RIC-COR study protocol. RIPC = remote ischaemic preconditioning.

The protocol for end-point acquisition and assessment is illustrated in Fig. 1.

### 2.8 Assessment of physiological responses of the coronary artery and microcirculation

All vasodilator therapy apart from sublingual glyceryl trinitrate was withheld for 24 h prior to coronary reactivity testing. Coronary angiograms were acquired using cardiac catheter laboratory X-ray (Innova®, GE Healthcare; Chicago, Illinois) and information technology equipment (Centricity®, GE Healthcare).

Reactivity testing of the coronary circulation, including the epicardial artery and its microvascular branches, included assessments of endothelial-dependent vasodilation following intracoronary infusion of graded doses of acetylcholine, and then assessment of endothelial-independent vasodilation following intracoronary administration of glyceryl trinitrate.

Endothelial function testing was performed using a standardised protocol by an operator blinded to the group allocation [36]. A 3 French infusion catheter (Cook Medical; Bloomington, Indiana) was placed in the proximal-to-mid segment of the epicardial coronary artery. A control intracoronary infusion of a 0.9% saline (2 ml over 2 min) was followed by intracoronary acetylcholine in incremental concentrations (10^−6^, 10^−5^, 10^−4^ mol/l). The infusion rate of acetylcholine was 2 ml/min giving approximate doses of 0.364, 3.64, and 36.4 μg, respectively.

Following each intracoronary infusion, an assessment of patient symptoms, 12‑lead ECG, and angiography using identical imaging projections (Innova®, GE Healthcare) and information technology equipment (Centricity®, GE Healthcare) were performed. Acetylcholine infusion was discontinued if: i) second or third degree atrioventricular block occurred; ii) there was angiographic evidence of severe epicardial vasospasm (reduction in the epicardial diameter ≥ 75%); iii) there was evidence of severe microvascular spasm (angina and ST-segment deviation occurring in the absence of epicardial coronary diameter change ≥ 75%) [37]. If these criteria were not present, we administered the next dose of acetylcholine. Following a second wash-out infusion of 0.9% saline for a period of 2 min, endothelial-independent vasodilation was tested with an intracoronary bolus of glyceryl trinitrate (400 μg).

### 2.9 Invasive coronary angiography and analysis

Quantitative coronary angiography (QCA) was performed by three experienced cardiologists (P.C., A.B., C.B.) in the Glasgow Angiography Core Laboratory, using proprietary automated edge-detection software (Medis QAngio XA, Leiden, Netherlands). The angiographic images were calibrated to the coronary guide catheter size. The end-diastolic angiographic image demonstrating the best luminal contrast opacification was chosen for analysis. The coronary segment distal to the infusion catheter or the coronary segment demonstrating the most marked diameter change in the main epicardial vessel was analysed to determine the mean coronary artery luminal diameter for the selected segment. Analysts were blinded to the group assignment.

### 2.10 Mechanistic evaluation of circulating biomarkers of endothelial function

Venous blood was obtained from the contralateral arm pre- and then within 1 h post-RIPC or sham procedure. Venous blood was analysed for circulating molecules associated with endothelial vasodilator function (myeloperoxidase (MPO), interleukin-6 (IL-6), von Willebrand factor (vWF), asymmetric dimethylarginine (ADMA)) and endothelial fibrinolytic function (tissue plasminogen activator (t-PA)). Laboratory methods are described in the online-only supplement. Laboratory technicians were blinded to the group allocation.

### 2.11 Electrocardiography acquisition and analysis

#### 2.11.1 Acquisition

We acquired 12-lead ECGs (Centricity®, GE Healthcare) before and after each 2-minute intracoronary infusion of acetylcholine to assess for changes in cardiac conduction linked to autonomic nervous system activation.

#### 2.11.2 Analysis

ECGs were analysed in the Glasgow ECG Core Laboratory by a blinded analyst (P.McC.) for PR duration, QRS duration, corrected QT interval (QTc, defined as the QT interval corrected for heart rate using Bazett's formula), presence of atrioventricular block and ST-segment deviation.

### 2.12 Statistical analyses

#### 2.12.1 Sample size calculation

Our pilot data found the standard deviation of the percentage change in mean lumen diameter for the maximum dose of acetylcholine administered compared to control to be 14.6%. Based on a two-sample t-test, 25 patients per group were required to have 80% power at a 5% significance level to detect a mean between group difference of 12%. A net difference of 12% is consistent with previously published effective treatments which improve coronary artery function.

#### 2.12.2 Primary outcome

The pre-defined primary outcome was the mean percentage change in coronary artery luminal diameter from baseline to the maximum administered acetylcholine infusion, using an analysis of variance (ANOVA) model. An analysis of covariance (ANCOVA) model was used to assess for the influence of age and sex. Data are presented as mean (SD) or n (%) where appropriate. Fisher's exact test was used to compare the between group difference in the coronary vessel undergoing interrogation. Statistical analyses were performed by an independent biostatistician using R version 3.1.2 (R.Y. based in the Glasgow Clinical Trials Unit). All p values are two-sided and a p value < 0.05 was considered statistically significant.

#### 2.12.3 Secondary outcomes

Pre-specified secondary outcomes were intended to provide information on mechanisms of the physiological responses.

Secondary outcomes defined by quantitative coronary angiography analysis:

1. Mean percentage change in coronary luminal diameter from baseline following intracoronary glyceryl trinitrate (400 μg).
2. Coronary endothelial dysfunction (defined as a decrease in luminal diameter of > 20% following intracoronary acetylcholine).
3. Epicardial coronary artery spasm (defined as a reduction in coronary luminal diameter > 90% following intracoronary acetylcholine [38].

Secondary outcomes defined by electrocardiography:

4. Microvascular spasm (defined as the occurrence of angina with ischaemic ST-segment ECG changes in the absence of epicardial coronary vasospasm (≤ 90% coronary luminal diameter reduction) [37], [39].
5. Presence and extent of ST-segment deviation (ST-segment elevation or depression).
6. Occurrence of atrioventricular block.

Secondary outcomes determined by immunoassays:

7. Circulating molecules reflecting endothelial function from baseline to up to 2 h.

## 3 Results

### 3.1 Study population

**Fig. 2:**
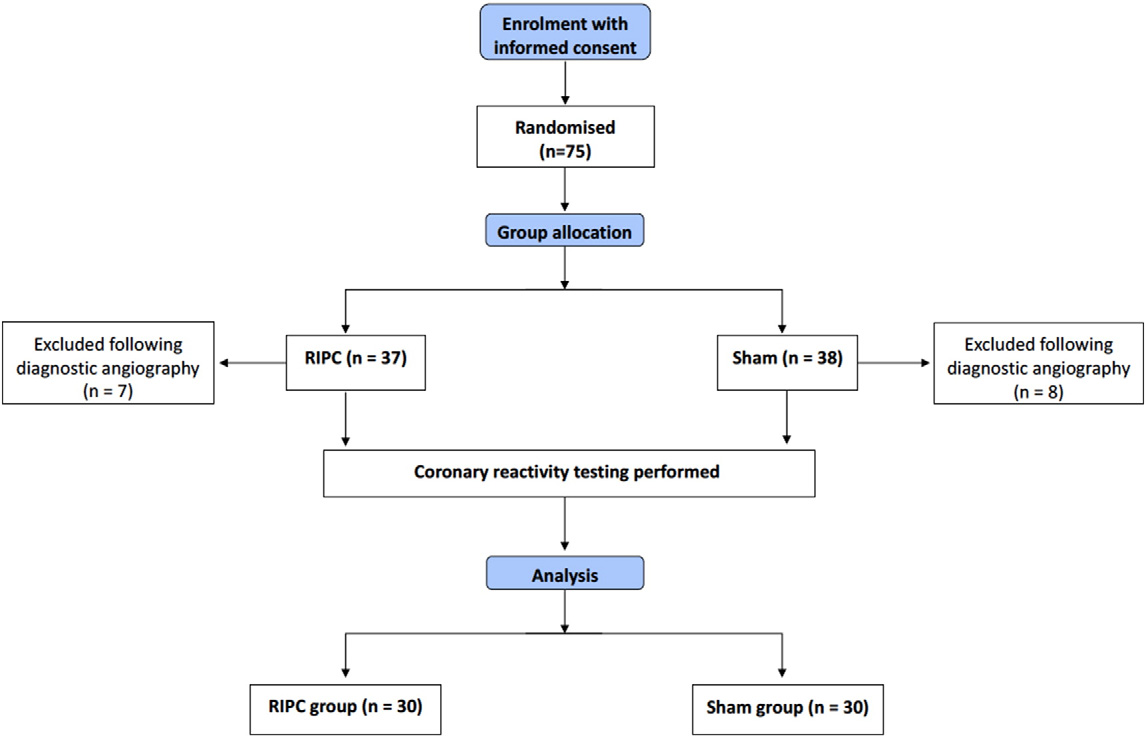
CONSORT (Consolidated Standards of Reporting Trials) flow diagram. RIPC = remote ischaemic preconditioning.

Seventy-five patients provided written informed consent and were randomised to RIPC or sham procedure (Table 1 and Fig. 2). Following diagnostic coronary angiography, 60 patients completed the study protocol (RIPC group n = 30, sham group n = 30). RIPC and sham procedures were well-tolerated (100% compliance), and no serious adverse events (SAEs) occurred. The left anterior descending, circumflex and right coronary arteries were evaluated in 13 (22%), 20 (33%), and 27 (45%) patients, respectively, with similar distributions between the groups (p = 0.79). In 15 patients, the coronary reactivity testing protocol was not performed (RIPC group (n = 7), sham group (n = 8) for the following reasons: no suitable vessel for coronary reactivity testing (n = 7), complications relating to diagnostic coronary angiography (n = 4), inability to maintain a stable intracoronary infusion catheter position (n = 3), coronary artery dissection (n = 1). Of the remaining 60 patients, 30 (100%) in the RIPC group and 27 (90%) in the sham group completed the coronary reactivity testing protocol. Three patients, all in the sham group, met the pre-specified criteria to discontinue the acetylcholine infusion before completing the protocol, for the following reasons: severe epicardial spasm (n = 2) and second degree atrioventricular block (n = 1).

**Table 1:** Baseline clinical characteristics.

Variable	Sham (n = 30)	RIPC (n = 30)	p-value
Age, years	57.8 (9.6)	57.2 (7.4)	0.79
Male sex	25 (83.3%)	23 (76.7%)	0.75
BMI, kg/m^2^	29.5 (5.0)	29.4 (3.9)	0.91
Blood pressure, mm Hg			
- Systolic	131.8 (18.5)	137.1 (21.8)	0.31
- Diastolic	70.4 (9.2)	75.9 (11.4)	0.04

*Medical history*			
Hypercholesterolaemia	16 (53.3%)	22 (73.3%)	0.18
Diabetes Mellitus	5 (16.7%)	6 (20.0%)	0.99
Current smoker	6 (20.0%)	8 (26.7%)	0.76
Hypertension	13 (43.3%)	21 (70.0%)	0.06
Family history of CAD	21 (70.0%)	19 (63.3%)	0.78
Prior myocardial infarction	8 (26.7%)	7 (23.3%)	0.99
Prior PCI	5 (16.7%)	4 (13.3%)	0.99
Cerebrovascular Disease	0 (0.0%)	1 (3.3%)	0.99
Renal dysfunction (eGFR < 60 ml/min)	2 (6.7%)	2 (6.7%)	0.99
Baseline drug therapy			
- Aspirin	28 (93.3%)	25 (83.3%)	0.42
- Clopidogrel	11 (36.7%)	9 (30.0%)	0.78
- Ticagrelor	2 (6.7%)	2 (6.7%)	0.99
- ACEi	16 (53.3%)	14 (46.7%)	0.80
- ARB	0 (0.0%)	3 (10.0%)	0.24
- Statin	27 (90.0%)	27 (90.0%)	0.99
- β-blocker	29 (96.7%)	22 (73.3%)	0.03
- Calcium-channel blocker	7 (23.3%)	12 (40.0%)	0.27
- Oral nitrate	10 (33.3%)	8 (26.7%)	0.78
- Nicorandil	3 (10.0%)	5 (16.7%)	0.71
Baseline mean coronary artery luminal diameter, mm	2.40 (0.53)	2.55 (0.54)	0.28

Data are presented as Mean (SD) or n (%) where appropriate. CAD = coronary artery disease, PCI = percutaneous coronary intervention, BMI = body mass index, ACEi = angiotensin converting enzyme inhibitor, ARB = angiotensin receptor blocker, eGFR = estimated glomerular filtration rate.

### 3.2 Primary outcome: coronary lumen diameter following intracoronary infusion of acetylcholine

The mean percentage change in coronary luminal diameter from baseline to the maximum administered acetylcholine dose was − 13.3 (22.3) and − 2.0 (17.2) % in the sham and RIPC groups, respectively (Table 2). There was a lower mean percentage reduction in coronary luminal diameter in the RIPC compared to sham group (mean difference 11.32%, 95% CI: 1.2 to 21.4, p = 0.032). This difference remained significant when accounting for the influence of age and sex by including these characteristics as covariates in an ANCOVA model (mean difference 11.01%, 95% CI: 1.01 to 21.0, p = 0.035).

**Table 2:** Primary and secondary outcomes.

Variable	Sham (n = 30) Mean (SD)	RIPC (n = 30) Mean (SD)	Between group difference in change from baseline Mean (CI)	p-value
*Coronary reactivity testing*				
Endothelial-dependent vasoreactivity testing (Mean coronary diameter at maximum ACh dose administered, mm)	2.08 (0.72)	2.50 (0.66)	11.32% (1.24, 21.40)	0.032
Endothelial-independent vasoreactivity testing (Mean coronary diameter following intracoronary GTN, mm)	2.59 (0.48)	2.78 (0.52)	1.18% (− 3.38, 5.75)	0.61

*Circulating cytokines implicated in endothelial function*				
MPO, ng/ml	Pre 3.7 (5.1) Post 3.4 (3.8)	Pre 3.8 (3.2) Post 2.9 (2.3)	− 0.5 (− 2.15, 1.15)	0.55
IL-6, pg/ml	Pre 3.5 (2.7) Post 2.8 (1.4)	Pre 3.4 (2.1) Post 3.0 (1.9)	0.32 (− 0.71, 1.36)	0.54
tPA, ng/ml	Pre 5.7 (1.7) Post 5.4 (2.2)	Pre 6.0 (2.0) Post 6.2 (2.6)	0.45 (− 0.24, 1.14)	0.20
vWF, U/ml	Pre 144.3 (24.8) Post 138.3 (26.4)	Pre 137.7 (26.6) Post 135.0 (32.0)	3.31 (− 4.58, 11.19)	0.41
ADMA, μmol/l	Pre 0.45 (0.08) Post 0.43 (0.09)	Pre 0.44 (0.09) Post 0.43 (0.09)	0.01 (− 0.03, 0.05)	0.57

*Electrocardiogram findings*				
PR duration, ms	9.6 (42.0)	0.4 (42.0)	− 9.2 (− 33.73, 15.33)	0.47
QRS duration, ms	− 1.8 (9.4)	− 1.3 (15.1)	0.54 (− 6.18, 7.26)	0.88
QTc duration, ms	4.7 (80.2)	− 26.6 (68.5)	− 31.32 (− 78.53, 15.9)	0.20
R-R interval, ms	− 7.0 (75.2)	6.5 (61.3)	13.57 (− 21.63, 48.77)	0.45

ACh = acetylcholine, GTN = glyceryl trinitrate, MPO = myeloperoxidase, IL-6 = interleukin-6, tPA = tissue plasminogen activator, vWF = von Willebrand factor.

### 3.3 Secondary outcomes

#### 3.3.1 Coronary reactivity testing secondary outcomes

There was no between group difference in endothelial-independent vasodilation in the RIPC compared to sham group (mean coronary luminal percentage diameter change 1.2%, 95% CI: − 3.4 to 5.8, p = 0.61) (Table 2). There was no statistically significant between group difference in the number of patients meeting the criteria for endothelial dysfunction (4 (13.3%) vs. 8 patients (26.7%), p = 0.33), epicardial vasospasm (0 (0.0%) vs. 1 patient (3.3%), p = 0.99), and microvascular spasm (4 (13.3%) vs. 3 patients (10.3%), p = 1.00), in the RIPC compared to sham group, respectively.

#### 3.3.2 Electrocardiogram findings

There were no between-group differences the ECG parameters (change from baseline in PR duration, QRS duration, QTc duration, or R-R interval; occurrence of atrioventricular block at the maximum administered dose of acetylcholine; ST-segment deviation).

#### 3.3.3 Circulating cytokines implicated in endothelial function

There were no differences in the circulating concentrations of markers of endothelial function in the RIPC compared to sham group (Table 2).

## 4 Discussion

We undertook a mechanistic, randomised, sham-controlled, blinded clinical trial of the effect of RIPC on endothelial function in the coronary circulation of patients with stable CAD. We have shown that RIPC performed immediately prior to invasive management results in a lower reduction in mean percentage coronary artery luminal diameter, consistent with enhanced coronary endothelial function. In other words, RIPC attenuated the extent of coronary vasoconstriction induced by intracoronary acetylcholine infusion. Secondly, RIPC did not result in increased circulating concentrations of several circulating cytokines that are implicated in endothelial vasodilator function (MPO, IL-6, vWF, ADMA) or in a marker of endothelial fibrinolytic function (t-PA). Thirdly, RIPC did not have an effect on cardiac conduction or on ST-segment deviation.

Our mechanistic results relating to RIPC and coronary endothelial function are novel and clinically relevant. RIPC had a moderate but tangible effect on coronary artery vasoconstriction (difference in mean percentage reduction in coronary luminal diameter 11.32%) in non-obstructed epicardial coronary arteries. Since the intervention was applied immediately before invasive management, it is transferable into clinical practice. Further, to minimise bias and enhance validity, the study design involved random assignment of treatment allocation, a sham procedure, researcher blinding, and independent statistical analysis.

The study participants all had stable CAD, and coronary endothelial dysfunction was prevalent. In normal coronary arteries, acetylcholine results in vasodilatation due to endothelial release of nitric oxide and resultant smooth muscle relaxation. In arteries with endothelial dysfunction, the direct action of acetylcholine on muscarinic smooth muscle receptors is unopposed and results in vasoconstriction [40]. In both groups of patients, a reduction in coronary artery diameter occurred with the maximum dose of acetylcholine administered, indicative of endothelial dysfunction co-existent with the underlying atherosclerotic CAD. However, the percentage change in mean coronary luminal diameter was lower with RIPC compared to sham i.e. there was less vasoconstriction with RIPC compared to the sham procedure.

Whether the cardioprotective mechanism induced by RIPC involves enhanced coronary blood flow is uncertain. Data from animal models and healthy human volunteers suggest that resting coronary blood flow may be enhanced by RIPC [41], [42]. Hoole et al. demonstrated that RIPC does not reduce coronary microvascular resistance, as assessed invasively using coronary thermodilution and Doppler flow velocity techniques, or enhance resting or hyperaemic coronary Doppler flow velocity [43]. In 54 patients with single and multi-vessel CAD undergoing elective PCI, there was no change in the index of microcirculatory resistance (IMR) or Doppler-derived microvascular resistance following both RIPC or cardiac ischaemic preconditioning [44], [45]. Our data indicate that RIPC alleviates vasoconstriction in atherosclerotic coronary arteries via an endothelial-dependent vasodilator mechanism. This result is concordant with other evidence suggestive of a beneficial effect of RIPC on peripheral endothelial function [23]. In animal models, ischaemic preconditioning prevents endothelial dysfunction following ischaemia-reperfusion injury [46]. In 30 patients undergoing invasive management of stable CAD, Lanza et al. demonstrated that invasive coronary angiography was associated with an impairment of peripheral endothelial function, and that RIPC prevented this detrimental effect [31]. For the first time, we extend this observation to the coronary circulation. Hoole et al. demonstrated in 242 patients with stable CAD undergoing elective PCI, a lower peri-procedural cardiac troponin release and reduced major adverse cardiac events at 6 months and 6 years follow-up [8], [47]. It is plausible that less coronary vasoconstriction, secondary to enhanced endothelial function induced by the RIPC stimulus, may result in better maintenance of myocardial perfusion (i.e. reduced ischaemia) and therefore less ischaemic injury in the heart. However, we did not demonstrate a between-group difference in ST-segment deviation during endothelial function testing. It is plausible that the serial ECGs lacked sensitivity for changes in myocardial perfusion secondary to RIPC, whereas a non-invasive stress test imaging earlier in the ischaemic cascade, such as stress perfusion cardiac magnetic resonance imaging, may have detected changes in myocardial perfusion.

In general, RIPC has been associated with reduced infarct size in proof-of-concept clinical studies [8], [9], [10], [11], [17]. It is plausible that one mechanism by which RIPC acts to reduce infarct size is to modify functional microvascular obstruction by altering the balance between pathological vasoconstriction and vasodilation and thus increasing myocardial blood flow [48]. Whether the attenuation in infarct size is sufficient to translate to improved clinical outcomes in patients following acute MI is currently being assessed in the CONDI-2 and ERIC phase 3 clinical trials. These pivotal trials are currently enrolling and are sufficiently large (overall sample size > 4000 patients) to provide conclusive results on the potential clinical benefits of RIPC in STEMI. In other settings, such as surgical revascularisation, RIPC has not been associated with improved health outcomes [49], [50], [51].

Experimental studies suggest that the mechanism of RIPC is similar to that of ischaemic preconditioning and the mechanistic pathway which conveys the cardioprotective signal is likely to involve both neural and humeral responses [21], [23]. We did not identify a circulating humoral factor derived from the vascular endothelium that may have mediated the favourable effects of RIPC on coronary artery function. There were no between-group differences in R-R interval, implying that a neural mechanism may not mediate the coronary vasoactive effects of RIPC. Further studies are warranted to assess the role of the sympathetic nervous system in RIPC.

Ischaemic preconditioning was first described in 1986 [1], and has been investigated in acute STEMI (type 1 MI), elective PCI (to reduce type 4a MI), and surgical coronary revascularisation (to reduce type 5 MI) [52]. Our randomised, controlled trial has identified RIPC-mediated effects on coronary artery function as a potential mechanism that may contribute to RIPC cardioprotection. The mechanisms involved in RIPC are the subject of intense research (e.g. the Consortium for preclinicAl assESsment of cARdioprotective therapies (CAESAR) [53]. Whether the favourable effect of RIPC on coronary artery function might translate into patient benefits, such as improved prognosis following acute MI, will be explored in current trials.

## 5 Limitations

Although patients were not blinded to their treatment group assignment, the primary and secondary outcomes were independent of any subjective responses from the participants. We undertook coronary reactivity testing in a single coronary artery, selected based on feasibility for instrumentation and epicardial disease characteristics. Although the sample size is limited, our study was prospectively designed and adequately powered to assess for between-group differences in the primary outcome. The statistical analysis was performed by a biostatistician independent of the research group. The statistical analysis with ANCOVA was adopted to allow for variations in endothelial function that might be related to between-patient differences in the extent and nature of vascular risk factors [36].

There was a statistically significant difference in the resting diastolic blood pressure (70.4 vs. 75.9 mm Hg in the sham and RIPC groups respectively), however there was no difference in systolic blood pressure or in the between-group distribution of a history of arterial hypertension. There was a statistically significant difference in the number of patients taking regular β-blocker therapy at baseline (29 versus 22 patients in the sham and RIPC groups respectively). β-blocker therapy may itself be cardioprotective, may attenuate the cardioprotective effect of RIPC [54], and may enhance endothelial function. Therefore, it may be hypothesised that we may have observed an even greater extent of enhanced coronary endothelial function had there been no between-group difference in β-blocker therapy at baseline.

We assessed coronary vasoreactivity with intracoronary acetylcholine and QCA as first described by Ludmer et al. in 1986 [40]. This technique allows assessment of coronary epicardial and microvascular endothelial-dependent and -independent vasodilation [39]. In contrast, simultaneous coronary blood flow assessment would have required use of an intracoronary Doppler guidewire. In order to minimise instrumentation of normal or minimally-diseased coronary arteries, and in the interests of patient safety, we did not use this method. Invasive indices of coronary microvascular resistance and vasodilatory capacity were not measured since prior studies have found that RIPC did not affect coronary resistance [43]. In this regard, QCA of the mean coronary artery lumen diameter is a reliable and sensitive parameter.

We did not collect coronary venous blood since additional instrumentation with right heart catheterisation may lead to patient discomfort and risk. Nor did we use pharmacological agents, such as endothelin or a nitric oxide synthase inhibitor, which could provoke sustained vasoconstriction. There were no SAEs related to the coronary reactivity testing in this study.

We did not assess cardiac biomarkers as this was a mechanistic study, and the effect of RIPC on surrogate cardiac outcomes e.g. troponin post-PCI, has already been described [8]. For logistical reasons we did not record heart rate variability, which may have provided further insights into autonomic nervous system activation.

## 6 Conclusions

We have performed a randomised, sham-controlled, single-blind clinical trial that for the first time provides a fundamental new insight into the mechanisms by which RIPC may have meaningful cardioprotective effects; namely by enhancing coronary artery endothelial function in patients with stable CAD.

## Funding

This work was supported by a Chest Heart & Stroke Scotland project grant award (R11/A136). D.C. is supported by a British Heart Foundation (BHF) Clinical Research Training Fellowship [FS/14/15/30661]. B.H. is supported by a BHF project grant [PG/14/97/31263]. P.McC. is supported by a National Institute for Health Research (NIHR) Efficacy and Mechanism Evaluation Programme (12/170/45). C.B. is supported by a BHF Centre of Research Excellence Award (RE/13/5/30177).

## Conflict of interest

There are no relevant conflicts of interest.

## Appendix A Supplementary data

Supplementary data: Laboratory methods; Figure 3. Coronary endothelial function testing results; Figure 4. Coronary reactivity testing results; Figure 5. RIC-COR study patient case example. Image 1
