# Supplementary material for: The effects of remote ischaemic preconditioning on coronary artery function in patients with stable coronary artery disease
Source: Int J Cardiol. 2018 Feb 1;252:24–30. doi: 10.1016/j.ijcard.2017.10.082 (PMC5761717; doi:10.1016/j.ijcard.2017.10.082)
Supplement: Supplementary file 1 — Supplementary data: Laboratory methods; Figure 3. Coronary endothelial function testing results; Figure 4. Coronary reactivity testing results; Figure 5. RIC-COR study patient case example. [file mmc1.docx]

# Online-only supplement

## Mechanistic evaluation of circulating biomarkers of endothelial function

Serum MPO and high sensitivity IL-6 were measured using commercially available ELISA kits (R&D systems, Oxon, UK) and using manufacturer quality control material. VWF antigen and tPA were measured in citrated plasma using commercially available ELISA kits (Asserachrom, Stago, Theale, UK) using the manufacturers quality control material. Serum ADMA was measured using commercially available ELISA kits (Immundiagnostik, Biosystems, Oxon, UK) using manufacturer quality control. All assays were performed according to manufacturer instructions.

## Figure Legends

**Figure 3.** Coronary endothelial function testing results.

**Figure 4.** Coronary reactivity testing results.

**Figure 5.** RIC-COR patient case example. A 55-year-old male presented with chronic stable angina (Canadian Cardiac Society class 3). Exercise electrocardiography testing was symptomatically and electrically positive in stage 1 of a full Bruce protocol. Diagnostic invasive coronary angiography demonstrated significant mid-apical left anterior descending artery (LAD) coronary artery stenosis. The right coronary artery (RCA) was unobstructed. Following multi-disciplinary team meeting, the patient was referred for percutaneous coronary intervention (PCI). The patient provided informed consent and was enrolled into the RIC-COR clinical trial. The patient was randomised to sham procedure. Prior to PCI to the LAD stenosis, coronary reactivity testing was performed in the RCA as per the RIC-COR protocol. On the baseline angiographic image, there is no obstructive stenosis within the RCA (A). At the maximum dose of acetylcholine infusion (10^-4^ mol/L), diffuse epicardial spasm is noted in the distal RCA (B, red arrows). This resolved following discontinuation of the acetylcholine infusion and administration of an intracoronary bolus of glyceryl trinitrate (400 µg) (C).

## Figure 3.

##
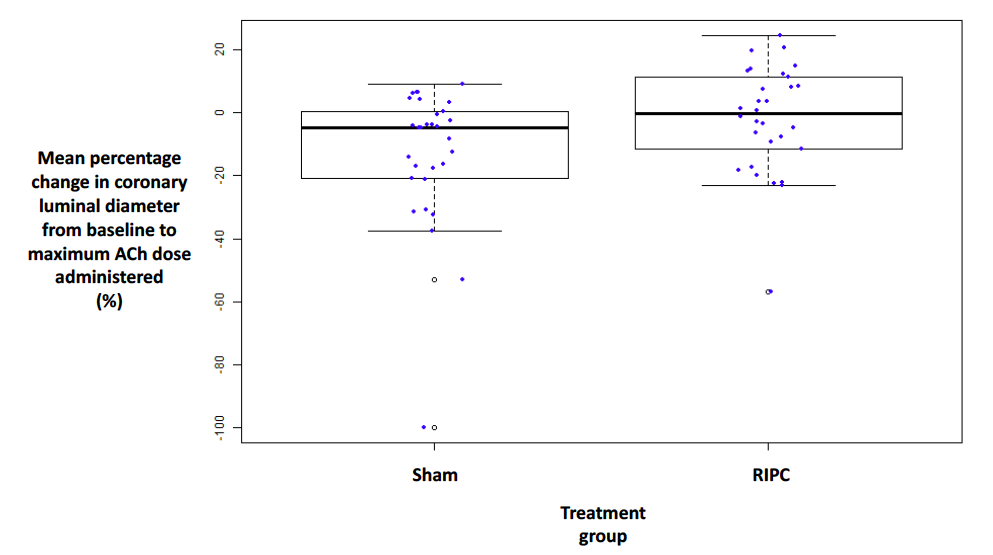
RIPC=remote ischaemic preconditioning, ACh=acetylcholine.

## Figure 4.


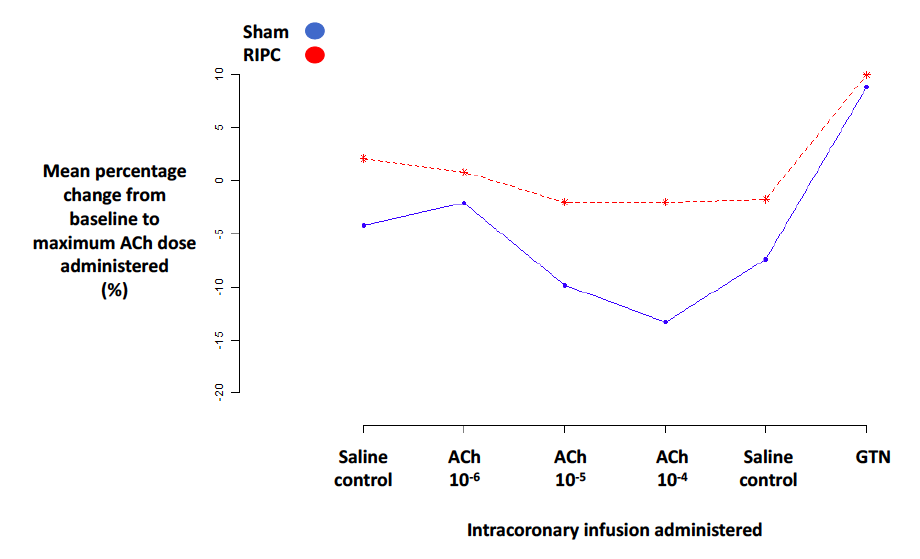


## RIPC=remote ischaemic preconditioning, ACh=acetylcholine, GTN=glyceryl trinitrate.

## Figure 5.

**
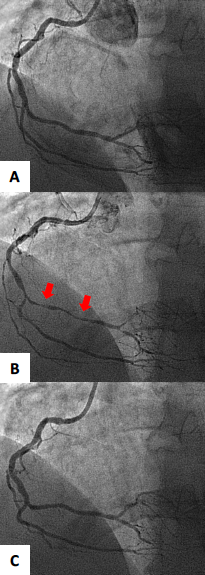
**
